# Evaluation of Neutralizing Antibodies Against Highly Pathogenic Coronaviruses: A Detailed Protocol for a Rapid Evaluation of Neutralizing Antibodies Using Vesicular Stomatitis Virus Pseudovirus-Based Assay

**DOI:** 10.3389/fmicb.2020.02020

**Published:** 2020-09-04

**Authors:** Sarah A. Almahboub, Abdullah Algaissi, Mohamed A. Alfaleh, M-Zaki ElAssouli, Anwar M. Hashem

**Affiliations:** ^1^Vaccines and Immunotherapy Unit, King Fahd Medical Research Center, King Abdulaziz University, Jeddah, Saudi Arabia; ^2^Department of Medical Laboratories Technology, College of Applied Medical Sciences, Jazan University, Jazan, Saudi Arabia; ^3^Medical Research Center, Jazan University, Jazan, Saudi Arabia; ^4^Faculty of Pharmacy, King Abdulaziz University, Jeddah, Saudi Arabia; ^5^Department of Medical Microbiology and Parasitology, Faculty of Medicine, King Abdulaziz University, Jeddah, Saudi Arabia

**Keywords:** coronaviruses, Middle East respiratory syndrome coronavirus, severe acute respiratory syndrome coronavirus 2, serological assay, vesicular stomatitis virus pseudovirus

## Abstract

Emerging highly pathogenic human coronaviruses (CoVs) represent a serious ongoing threat to the public health worldwide. The spike (S) proteins of CoVs are surface glycoproteins that facilitate viral entry into host cells via attachment to their respective cellular receptors. The S protein is believed to be a major immunogenic component of CoVs and a target for neutralizing antibodies (nAbs) and most candidate vaccines. Development of a safe and convenient assay is thus urgently needed to determine the prevalence of CoVs nAbs in the population, to study immune response in infected individuals, and to aid in vaccines and viral entry inhibitor evaluation. While live virus-based neutralization assays are used as gold standard serological methods to detect and measure nAbs, handling of highly pathogenic live CoVs requires strict bio-containment conditions in biosafety level-3 (BSL-3) laboratories. On the other hand, use of replication-incompetent pseudoviruses bearing CoVs S proteins could represent a safe and useful method to detect nAbs in serum samples under biosafety level-2 (BSL-2) conditions. Here, we describe a detailed protocol of a safe and convenient assay to generate vesicular stomatitis virus (VSV)-based pseudoviruses to evaluate and measure nAbs against highly pathogenic CoVs. The protocol covers methods to produce VSV pseudovirus bearing the S protein of the Middle East respiratory syndrome-CoV (MERS-CoV) and the severe acute respiratory syndrome-CoV-2 (SARS-CoV-2), pseudovirus titration, and pseudovirus neutralization assay. Such assay could be adapted by different laboratories and researchers working on highly pathogenic CoVs without the need to handle live viruses in the BSL-3 environment.

## Introduction

Coronaviruses (CoVs) are the largest group of enveloped positive-sense RNA viruses that primarily infect the respiratory and gastrointestinal tracts of birds and mammals (Fehr and Perlman, 2015). Many CoVs are zoonotic viruses that are capable of crossing the species barrier and infecting other hosts including humans (Kandeel et al., 2020). Human CoVs mainly cause mild respiratory tract infections, and no highly pathogenic CoVs were recognized until the beginning of the twenty-first century (Cui et al., 2019). Since 2002, a number of highly pathogenic human CoVs have emerged including the severe acute respiratory syndrome-CoV (SARS-CoV) in 2002/2003 and the Middle East respiratory syndrome-CoV (MERS-CoV) in 2012 (Ksiazek et al., 2003; Zaki et al., 2012). In December 2019, a novel human highly pathogenic CoV known as SARS-CoV-2 has emerged in Wuhan, China, causing the coronavirus disease 2019 (COVID-19) pandemic (Wu et al., 2020; Zhu et al., 2020). While SARS-CoV has disappeared, both MERS-CoV and SARS-CoV-2 continue to be a major global threat especially that to date no clinically proven treatments or vaccines are available for human use (Al-Amri et al., 2017; Liu et al., 2018; Ma et al., 2020; Padron-Regalado, 2020; Tse et al., 2020).

The spike (S) proteins of CoVs are surface glycoproteins that facilitate viral entry into host cells. The S1 subunit at the N terminal end of the S protein contains the receptor-binding domain (RBD) responsible for the attachment to cellular receptors, while the S2 subunit at the C-terminus mediates the fusion with the host membranes. The S protein of SARS-CoV and SARS-CoV-2 binds to the angiotensin-converting enzyme 2 (ACE2) as a cellular receptor, while the S protein of MERS-CoV utilizes the dipeptidyl peptidase 4 (DPP-4) (Liu et al., 2018; Hoffmann et al., 2020). The S protein, particularly the RBD, is considered as a major immunogenic component of CoVs and a target for most neutralizing antibodies (nAbs).

Although live virus-based neutralization methods are the gold standard serological assays to detect and measure nAbs levels, they require working under strict bio-containment conditions in biosafety level-3 (BSL-3) laboratories when working with highly pathogenic CoVs (Algaissi and Hashem, 2020). Other serological assays such as conventional enzyme-linked immunosorbent assay (ELISA) and immunofluorescence assay (IFA) have been utilized for CoV antibody screening; however, cross-reactivity with other common CoVs may lead to false-positive results (Lester et al., 2019; Degnah et al., 2020). Additionally, positivity in these assays does not necessarily reflect the presence of nAbs in samples, requiring other confirmatory functional bioassays. Thus, replication-incompetent pseudotyped viruses bearing S proteins from highly pathogenic CoVs could represent an alternative safe and convenient method for CoVs nAb detection and quantification in serum samples under biosafety level-2 (BSL-2) conditions (Almasaud et al., 2020). Several reports have shown encouraging results by utilizing vesicular stomatitis virus (VSV) as a platform to generate pseudoviruses that can be used in seroepidemiological studies, vaccine development, monoclonal antibodies and entry inhibitors screening, and basic research investigations of CoVs (Fukushi et al., 2005; Lester et al., 2019; Nie et al., 2020).

VSV is a zoonotic enveloped negative-stranded RNA virus that infects a wide range of animals and less frequently humans causing mild flu-like illness symptoms (Rodriguez, 2002; Tani et al., 2011). The small genome (11 kb), simple structure, and ability to grow in different types of mammalian cells with high titer made VSV a promising virus vector and a valuable tool in molecular biology and virology fields (Ruedas and Connor, 2017). Interestingly, recombinant VSV (rVSV) with G gene being replaced by reporter luciferase gene (rVSV-ΔG-luciferase) can normally bud from cells transfected with mammalian expression plasmid encoding VSV G protein or heterologous surface protein from other viruses (Whitt, 2010; Tani et al., 2011). Thus, rVSV-ΔG-luciferase system could be used to produce single-round replication-incompetent VSV pseudoviruses bearing any viral surface glycoprotein especially from those requiring work under BSL-3 and BSL-4 containments in BSL-2 laboratories (Whitt, 2010).

**FIGURE 1:**
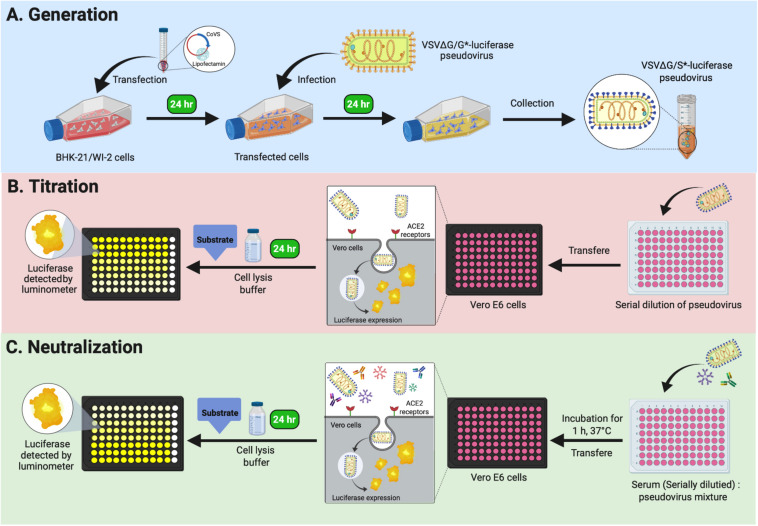
Graphical overview of Protocols 1, 2, and 3. **(A)** Protocol 1: generation of VSV pseudoviruses bearing CoV S protein. **(B)** Protocol 2: titration assay of the generated VSV pseudoviruses. **(C)** Protocol 3: neutralization assay to determine CoV-specific nAb titers in serum samples. VSV, vesicular stomatitis virus; CoV, coronavirus; nAb, neutralizing antibody.

In these detailed protocols, we explain step by step how to generate VSV pseudoviruses bearing S proteins from MERS-CoV and SARS-CoV-2 using transient expression in BHK-21/WI-2 cells (Protocol 1). This is followed by pseudovirus titration method (Protocol 2) and pseudovirus-based neutralization assay (Protocol 3) relying on reading quantitative luciferase luminescence signals. We utilized this system to conduct seroprevalence studies and measure nAbs in MERS-CoV and SARS-CoV-2 infected patients. Notes and comments have been added to overcome any difficulties. We believe that the described platform (Figure 1) can be adapted and used for research studies as well as diagnostic purposes.

## Materials and Methods

The detailed protocols described here can serve as convenient methods to detect MERS-CoV and SARS-CoV-2 nAbs in serum samples under BSL-2 conditions. The pseudovirus assays detailed here could also be used to evaluate the immunogenicity of vaccines and potency of monoclonal antibodies, other biologics, and small molecules. These assays rely on a well-established technique using rVSV-ΔG/G^∗^-luciferase pseudovirus system. Most reagents required for this system are commercially available and could be adopted and used by researchers and laboratories around the world. Such assays provide a number of advantages over standard serological assays including ability to evaluate nAbs in serum samples under BSL-2 conditions with minimal equipment and relatively low cost.

### Materials and Equipment

– Baby Hamster kidney BHK-21/WI-2 cell line (Kerafast catalog # EH1011).
– African Green monkey kidney-derived Vero E6 cell line [American Type Culture Collection (ATCC) catalog # 1586; Huh-7 cells could be used instead of Vero E6 cells].
– Dulbecco’s modified Eagle’s medium with L-glutamine (DMEM; BioWhittaker catalog #12-604F) supplemented with 5% fetal bovine serum (i.e., DMEM-5).
– Opti-MEM^®^ I (1 ×) reduced-serum medium (Gibco catalog # 31985062 or equivalent).
– 1 × phosphate-buffered saline (PBS) (Biosera catalog # LM-S2041 or equivalent).
– 1 × trypsin (Gibco catalog # 12484-028 or equivalent).
– pcDNA-S: Mammalian expression vector expressing MERS-CoV or SARS-CoV-2 S protein.
– pCAGGS-G-Kan plasmid (Kerafast catalog # EH1017).
– Lipofectamine^TM^ 2000 transfection reagent (Invitrogen catalog # 11668019; other transfection reagents or methods could be used as alternatives if available).
– rVSV-ΔG/G^∗^-luciferase (Kerafast catalog # EH1020-PM).
– Rabbit anti-VSV-G polyclonal antibodies (commercial or in-house made; other species could be used to generate antibodies).
– 5 × cell culture lysis reagent (CCLR) (Promega catalog # E1531).
– Luciferase assay system (Promega catalog # E1501).
– Serum samples.
– 96-well cell culture plate (white or black plate with clear bottom, COSTAR or equivalent).
– U-shaped 96-well cell culture plate (SPL Life Sciences catalog # 30096 or equivalent).
– Sterile reservoirs.
– Tissue culture T175 flasks with vented caps (SPL Life Sciences catalog # 71175 or equivalent).
– Sterile microcentrifuge tubes.

– Polypropylene sterile conical tubes:
  15 ml (Falcon catalog # 352099 or equivalent).
  50 ml (Falcon catalog # 352098 or equivalent).

– Sterile serological pipettes.

5 ml (SPL Life Sciences # 91005 or equivalent).
10 ml (SPL Life Sciences # 91010 or equivalent).
25 ml (SPL Life Sciences # 91025 or equivalent).

– Micropipettes.
– Micropipette tips.

10-μl filter tips, low retention (BIOLOGIX catalog # 23-0011 or equivalent).
200-μl filter tips, low retention (BIOLOGIX catalog # 23-0201 or equivalent).
1,000-μl filter tips, low retention (BIOLOGIX catalog # 23-1001 or equivalent).

– BSL-2 cabinet.
– Cell counter hemocytometer or equivalent.
– 37°C incubator with CO_2_ (Heal Force HF90 or equivalent).
– Water bath (Lab Tech LWB-111D or equivalent).
– Benchtop low-speed centrifuge (Sigma 2-16P or equivalent).
– Inverted microscope (Olympus CK30 or equivalent).
– Timer.
– Ultra-low freezer (-80°C).
– 70% ethanol; to sanitize all materials before use it inside the BSL-2 cabinet.

### Methods

#### Protocol 1: Production of Vesicular Stomatitis Virus Pseudoviruses Bearing Coronavirus S Protein

This protocol can be used to generate VSV pseudoviruses bearing S protein (rVSV-ΔG/S^∗^-luciferase) from either MERS-CoV or SARS-CoV-2 in BHK-21/WI-2 cell line (Figure 1A). Full-length codon-optimized MERS-CoV (GenBank accession number: KF958702) or SARS-CoV-2 (GenBank accession number: MN908947) S genes were synthesized and cloned into pcDNA3.1 mammalian expression vector. Genes were cloned into the vector following standard cloning techniques. Plasmids were transformed into DH5α cells or similar cells using ampicillin as a selection marker. High-quality purified plasmids were obtained using commercially available endotoxin-free maxiprep kit. A pCAGGS expression plasmid encoding VSV glycoprotein (pCAGGS-G) was used to generate rVSV-ΔG/G^∗^luciferase, which was used as a positive control (PC) pseudovirus. Lipofectamine^TM^ 2000 transfection reagent was used to deliver the constructed plasmids into BHK-21/WI-2 cells to express the desired protein on the cells surface. In total, 3 days is needed to generate rVSV-ΔG/S^∗^-luciferase pseudovirus by using BHK-21/WI-2 cells at ∼70% confluency in a T175 tissue culture flask as described in the following steps.

**Day 1: BHK-21/WI-2 Cell Preparation**

1. In BSL-2 cabinet, passage T75 tissue culture flask of 90% confluent BHK-21/WI-2 cells at 1:4 ratio (∼3 × 10^6^ cells) into a T175 tissue culture flask using DMEM-5 to be ∼70% confluent the next day.
2. Incubate the cells overnight at 37°C in 5% CO_2_ humidified incubator.

**Day 2: BHK-21/WI-2 Cell Transfection With Expression Vector Encoding Coronavirus S Protein**

1. The next day, check the cells under the inverted microscope to ensure that they are healthy and at the required confluency.
2. Replace the growth medium of the plated BHK-21/WI-2 cells in the T175 tissue culture flask with 22 ml of pre-warmed DMEM-5. Return the flask to the incubator until the transfection mixture is prepared.
3. Add 1.75 ml of Opti-MEM reduced serum medium into two 15-ml sterile polypropylene conical tubes: tube A receives 46 μg of a mammalian expression vector expressing S protein from either MERS-CoV or SARS-CoV-2, while tube B receives 92 μl of Lipofectamine^TM^ 2000 transfection reagent. As a PC, a T175 flask of BHK-21/WI-2 cells can be transfected with expression plasmid encoding VSV-G (pCAGGS-G-Kan plasmid) to generate rVSV-ΔG/G^∗^luciferase (this stock can be used later as a PC in the titration assay or to replenish rVSV-ΔG/G^∗^luciferase stock).
4. Mix the mixture in each tube by pipetting up and down 10 times, and incubate them for 5 min at room temperature.
5. Transfer the plasmid mixture in tube A into tube B and mix gently by pipetting up and down 10 times.
6. Incubate the transfection mixture for 20 min at room temperature.
7. Take out the BHK-21/WI-2 cells in the T175 flask from the incubator and transfect the cells by adding the 3.5 ml transfection mixture dropwise on the cell monolayer using 5-ml sterile serological pipette while swirling the flask gently to ensure even dispersal.
8. Incubate the transfected tissue culture flask for 24 h at 37°C in 5% CO_2_ humidified incubator.

**Day 3: Infection of Transfected Cells With rVSV-Δ*G/G^∗^-Luciferase***

1. In a 15-ml polypropylene sterile tube, prepare the virus inoculation mixture by adding 5 ml of DMEM-5 containing an amount of rVSV-ΔG/G^∗^luciferase equivalent to multiplicity of infection (MOI) of 4 using the working stock virus from Kerafast or in-house produced rVSV-ΔG/G^∗^luciferase (see *Generation of rVSV-*Δ*G/MERS-S^∗^-Luciferase and rVSV-*Δ*G/SARS-2-S^∗^-Luciferase Pseudoviruses* for rVSV-ΔG/G^∗^luciferase titration).
2. Take out the T175 flask containing the transfected BHK-21/WI-2 cells from Day 2 experiment from the incubator, and remove the growth medium.
3. Infect the cells with the 5 ml of media containing the rVSV-ΔG/G^∗^luciferase, and make sure to distribute equally over the cell monolayer.
4. Incubate the cells for 1 h at 37°C in 5% CO_2_ humidified incubator, and distribute the virus by gently rocking the T175 flask every 10 min.
5. During the incubation time, dilute rabbit polyclonal anti-VSV-G antibodies at a 1:1,000 dilution in a 50-ml polypropylene sterile conical tube containing 15 ml of pre-warmed DMEM-5. Alternatively, 1 μg/ml of anti-VSV-G antibody (Kerafast Cat # EB0010) can be used.
6. After 1-h incubation, remove the virus inoculum and wash the cells twice with 12 ml of pre-warmed 1 × PBS.
7. Add the prepared 15 ml of DMEM-5 supplemented with anti-VSV-G antibodies to the cell monolayer. In case of generating rVSV-ΔG/G^∗^-luciferase stock, use DMEM-5 without anti-VSV-G antibodies.
8. Incubate the flask for 24 h at 37°C in 5% CO_2_ humidified incubator.

**Day 4: Collection and Storage of the Generated Vesicular Stomatitis Virus Pseudoviruses**

1. The next day, collect the supernatant that contains the VSV pseudoviruses in a 50-ml polypropylene sterile conical tube.
2. Remove the cells debris by centrifugation of the supernatant at 600 × g for 5 min.
3. Aliquot the clarified supernatant as 1 ml into appropriately labeled sterile microcentrifuge tubes.
4. Store the generated pseudoviruses stocks at -80°C.

#### Protocol 2: Titration Assay of the Generated Vesicular Stomatitis Virus Pseudoviruses by Measuring Luciferase Activity

**FIGURE 2:**
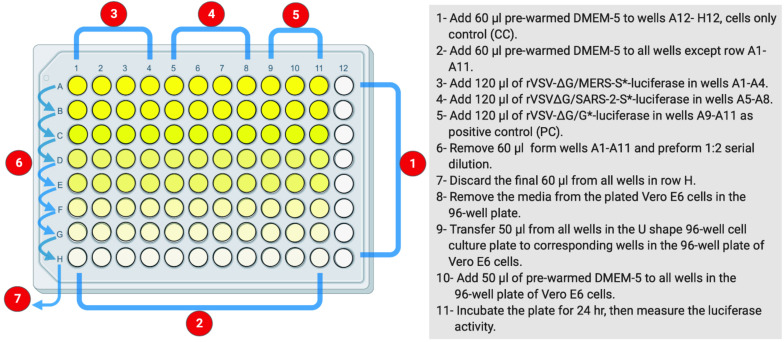
The layout of U-shaped 96-well cell culture plat for rVSV-ΔG/S*-luciferase pseudovirus titration in Protocol 2. The preparation steps are indicated in sequential numbers. rVSV, recombinant vesicular stomatitis virus.

This protocol is based on the use of luciferase activity as a main readout of the system to titrate the produced rVSV pseudoviruses (Figure 1B). The measured luciferase activity is defined as relative luminescence unit (RLU). This protocol is summarized in Figure 2. The protocol details are in the following steps.

**Day 1: Cell Preparation**

1. Count Vero E6 cells from confluent T75 tissue culture flask using a hemocytometer slide and trypan blue solution. It is preferred to subculture confluent Vero E6 at 1:4 ratio 48 h before use.
2. Prepare 11 ml of Vero E6 cells suspension at a density of 2 × 10^5^ cells/ml in a 50-ml polypropylene sterile conical tube using pre-warmed DMEM-5.
3. Seed the Vero E6 cells suspension in a 96-well white or black cell culture plate with clear bottom by distributing 100 μl of the cells/well using multichannel pipette, sterile filtered tips, and sterile reservoir. Every 100 μl should contain 2 × 10^4^ cells in total.
4. Incubate the seeded 96-well plate for 24 h at 37°C in 5% CO_2_ humidified incubator.

To save time, steps 1–4 can be done on Day 3 or 4 of Protocol 1.

**Day 2: Cell Infection With Generated Vesicular Stomatitis Virus Pseudoviruses***Bearing Coronavirus S Gene*

1. In a sterile U-shaped 96-well cell culture plate, add 60 μl of pre-warmed DMEM-5 to all wells in column 12 as a negative cell control; cell-only control (CC).
2. Add 60 μl of pre-warmed DMEM-5 to all wells in columns 1–11 in rows B to H using multichannel pipette, filtered tips, and sterile reservoir.
3. Thaw the supernatant containing generated VSV pseudotyped virus on ice.
4. Add 120 μl of the supernatant containing rVSV-ΔG/S^∗^-luciferase pseudovirus in wells A1 to A8. As an example, rVSV-ΔG/MERS-S^∗^-luciferase pseudovirus can be added in wells A1 to A4 and rVSV-ΔG/SARS-2-S^∗^-luciferase pseudovirus in wells A5 to A8.
5. As a PC, add 120 μl of the supernatant containing rVSV-ΔG/G^∗^-luciferase pseudovirus in wells A9 to A11.
6. Remove 60 μl from virus-containing wells in row A (A1–A11), and perform 1:2 serial dilution downward to all wells below using multichannel pipette and filtered tips. Other dilutions such as 1:3 or 0.5 log could be used.
7. During each dilution step, mix well by pipetting eight times up and down.
8. Continue the dilution until row H, and discard the final 60 μl from the last wells in row H.
9. Remove the media from the plated Vero E6 cells in the 96-well plate that was seeded on Day 1.
10. With the use of multichannel pipette and filtered tips, transfer 50 μl from all wells in the U-shaped 96-well cell culture plate to corresponding wells in the 96-well plate of Vero E6 cells.
11. Add 50 μl of pre-warmed DMEM-5 to all wells in the 96-well plate of Vero E6 cells.
12. Incubate the plate for 24 h at 37°C in 5% CO_2_ humidified incubator.

**Day 3: Luciferase Assay**

1. Prepare 1 × lysis buffer from 5 × CCLR in a 15-ml polypropylene sterile conical tube by adding four volumes of water to 1 volume of 5 × CCLR. A total of 2.5 ml of 1 × lysis buffer will be enough for each 96-well plate (20 μl/well).
2. By using Promega luciferase assay system (Promega catalog # E1501), prepare the luciferase assay reagent by adding 10 ml of luciferase assay buffer to a vial containing lyophilized luciferase assay substrate.
3. Remove the growth media from all wells in the 96-well cell culture plate of Vero E6 cells from Day 2.
4. Rinse the cells in all wells with 50 μl 1 × PBS, and make sure not to dislodge the cells. Ensure complete removal of any residual liquid.
5. Add 20 μl of the prepared 1 × cells lysis buffer to each well.
6. Add 50 μl of the prepared luciferase reagent to each well. Work with two columns each time to complete steps 5–7, which include cell lysis and addition of luciferase reagent, and measure the luciferase activity. Repeat cycle for the remaining columns. Alternatively, a luminometer supplied with two injectors for both lysis buffer and luciferase substrate can be used to facilitate the process by measuring light produced from the reaction ∼8 s after adding the substrate using an integration time of 5–30 s.
7. Measure the light produced for a period of ∼8 s using luminometer, and save the results. The reaction is nearly constant for about 1 min and then decays slowly, with a half-life of ~10 min. The typical delay time is 2 s, and the typical read time is 10 s.
8. Plot virus dilution vs. RLU readout to select the needed amount of virus for neutralization assay. Select a dilution that results in a signal above cell-only control and in the linear part of the curve.

#### Protocol 3: Neutralization Assay to Determine Coronavirus-Specific Neutralizing Antibody Titers in Serum Samples

**FIGURE 3:**
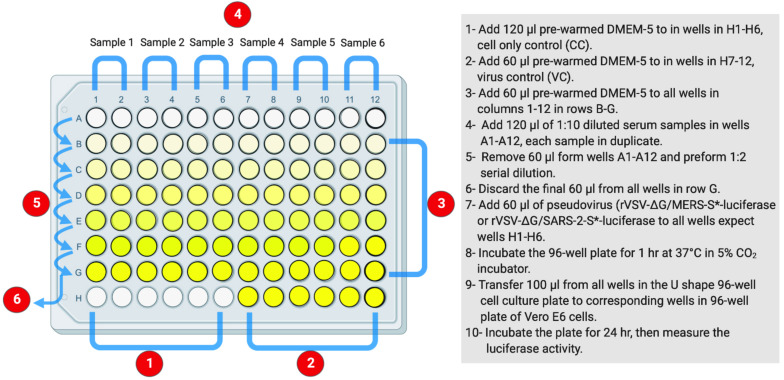
The layout of U-shaped 96-well cell culture plat for rVSV-ΔG/S*-luciferase pseudovirus neutralization assay in Protocol 3. The preparation steps are indicated in sequential numbers. rVSV, recombinant vesicular stomatitis virus.

As in Protocol 2, measuring of nAb titers depends on using luciferase-based assay. Inhibition of the generated pseudovirus entry into Vero E6 cells by nAbs is correlated with the decreased levels of luciferase expression signals. This assay could be used to measure nAb titers from different species including humans and animals as well as testing monoclonal antibodies. Figure 3 illustrates the workflow as it is described in the following steps.

**Day 1: Cell Preparation**

1. Seed 2 × 10^4^ Vero E6 cells per well in a 96-well white or black cell culture plate with clear bottom. This can be achieved by following steps 1–4 on Day 1 in Protocol 2 (*Protocol 2: Titration Assay of the Generated Vesicular Stomatitis Virus Pseudoviruses by Measuring Luciferase Activity*).

**Day 2: Pseudovirus Neutralization Assay**

1. In a sterile U-shaped 96-well cell culture plate, add 60 μl of pre-warmed DMEM-5 to all wells in columns 1–12 in rows B to G.
2. Add 120 μl of pre-warmed DMEM-5 to wells H1 to H6 to serve as negative cell control; CC.
3. Add 60 μl of pre-warmed DMEM-5 to wells H7 to H12 to be used as virus control (VC).
4. Add 120 μl of 1:10 dilution of heat-inactivated serum samples, at 56°C for 30 min, in wells in row A; add each sample in duplicate.
5. Remove 60 μl from serum-containing wells in wells A1–A12, and perform 1:2 serial dilutions downward to all wells below. Other dilutions such as 1:3 or 0.5 log could be used.
6. During each dilution step, mix well by pipetting eight times up and down.
7. Continue the dilution until row G, and discard the final 60 μl from the last wells in row G.
8. Prepare rVSV pseudovirus suspension at a concentration of 1 × 10^6^ RLU per ml. A total of 7.5 ml is needed for one 96-well plate.
9. Add 60 μl of pseudovirus suspension into each well in the plate except wells H1 to H6 (CC).
10. Incubate the plate for 1 h at 37°C in 5% CO_2_ humidified incubator.
11. Take out the plated Vero E6 cells in 96-well cell culture plate from the incubator that was seeded on Day 1, and remove the growth medium.
12. With the use of a multichannel pipette and filtered tips, transfer 100 μl from all wells in the U-shaped 96-well cell culture plate to corresponding wells in the 96-well plate of Vero E6 cells.
13. Incubate the plate for 24 h at 37°C in 5% CO_2_ humidified incubator.

**Day 3: Luciferase Assay**

1. Follow steps 1–7, which have been described in the luciferase assay on Day 3 in Protocol 2 (*Protocol 2: Titration Assay of the Generated Vesicular Stomatitis Virus Pseudoviruses by Measuring Luciferase Activity*) to measure luciferase activity.
2. Inhibition (%) of luciferase activity from each serum dilution could be calculated as follows: 100 - [(mean RLU from each sample (virus + serum) - mean RLU from CC)/(mean RLU from VC - mean RLU from CC) × 100].
3. Inhibition (%) should be plotted against each dilution using four-parameter logistic (4PL) curve, and 50% inhibitory concentration (IC_50_) values for each sample could be computed using graph prism or similar software.

## Results

### Generation of rVSV-ΔG/MERS-S^∗^-Luciferase and rVSV-ΔG/SARS-2-S^∗^-Luciferase Pseudoviruses

**FIGURE 4:**
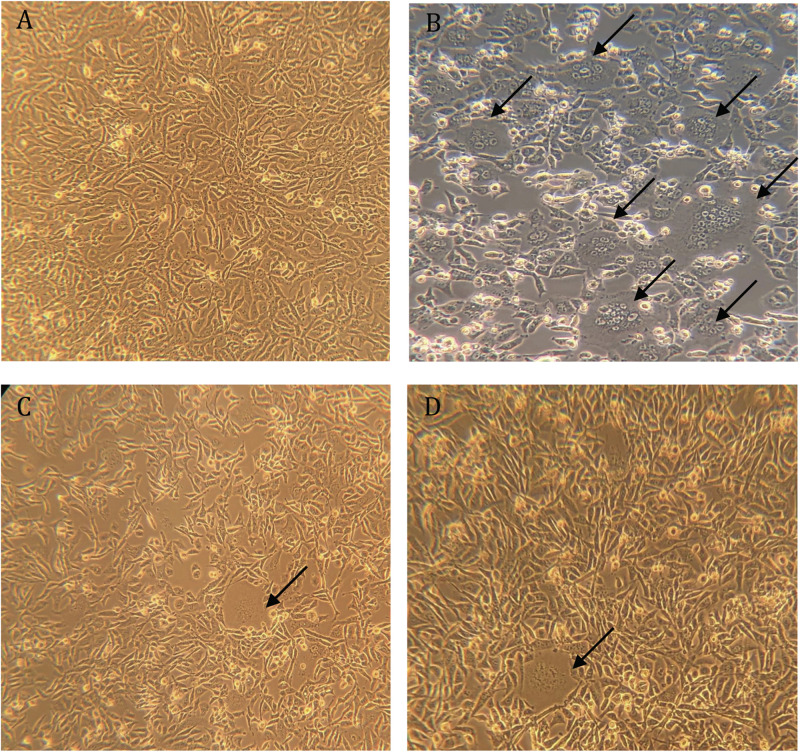
Syncytia formation in BHK-21/WI-2 cells 24 h post-transfection. **(A)** Cell control. **(B)** Cells transfected with pCAGGS-G. **(C)** Cells transfected with pcDNA-SARS-2-S. **(D)** Cells transfected with pcDNA-MERS-S. Arrows highlight the observed syncytia. Images are representative from three independent experiments.

The highly transfectable BHK-21/WI-2 cell line was selected to recover the rVSV pseudoviruses. BHK-21/WI-2 cells were initially transfected with pcDNA mammalian expression vector expressing the S protein of either MERS-CoV or SARS-CoV-2. We used pcDNA expressing MERS-CoV S to generate rVSV pseudovirus bearing MERS-CoV S (rVSV-ΔG/MERS-S^∗^-luciferase), and pcDNA expressing SARS-CoV-2 S is used to generate rVSV pseudovirus bearing SARS-CoV-2 S (rVSV-ΔG/SARS-2-S^∗^-luciferase). By examining the transfected cells 24 h post-transfection using inverted microscope, syncytia were found throughout the cell monolayer. They were more obvious in cells transfected with pCAGGS-G, which means that sufficient VSV-G protein has been expressed (Figure 4).

Different mammalian expression vectors such as pCAGGS, plasmid purification kits, or methods, or transfection reagents could be used (Grehan et al., 2015; Almasaud et al., 2020). Additionally, this system could be used to generate any pseudotyped viruses expressing the protein of interest simply by using other plasmids. It is recommended to generate additional working stocks of rVSV-ΔG/G^∗^-luciferase virus by amplifying an aliquot of the rVSV-ΔG/G^∗^-luciferase stock from Kerafast using BHK-21/WI-2 cells transfected with pCAGGS-G (expression plasmid encoding VSV-G protein) as described in step 3 on Day 2 in Protocol 1 (*Protocol 1: Production of Vesicular Stomatitis Virus Pseudoviruses Bearing Coronavirus S Protein*). Plaque assay can be used to determine rVSV-ΔG/G^∗^-luciferase titer, and calculate MOI needed for step 1 on Day 3 in Protocol 1 (*Protocol 1: Production of Vesicular Stomatitis Virus Pseudoviruses Bearing Coronavirus S Protein*) as previously described (Whitt, 2010).

**FIGURE 5:**
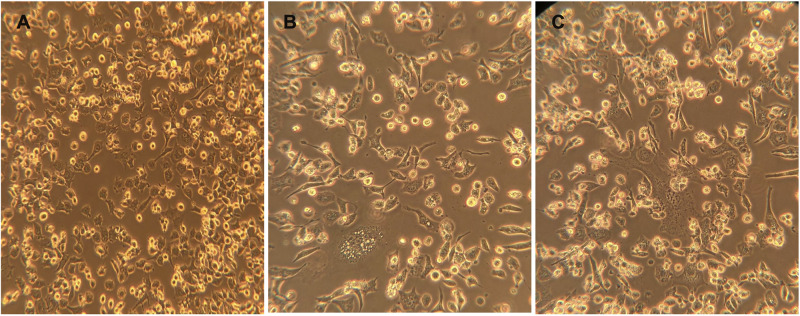
Cells rounding 24 h after infection with rVSV-ΔG/G*-luciferase pseudovirus. Cytopathic effect (cells rounding) of BHK-21/WI-2 cells transfected with **(A)** pCAGGS-G, **(B)** pcDNA-SARS-2-S, or **(C)** pcDNA-MERS-S and infected with rVSV-ΔG/G*-luciferase pseudovirus. Images are representative from three independent experiments. rVSV, recombinant vesicular stomatitis virus.

The transfected BHK-21/WI-2 cells with pcDNA/MERS-S, pcDNA/SARS-2-S, and pCAGGS-G were infected with rVSV-ΔG/G^∗^-luciferase stock after 24 h of the transfection. The pseudoviruses rVSV-ΔG/MERS-S^∗^-luciferase, rVSV-ΔG/SARS-2-S^∗^-luciferase, and the PC rVSV-ΔG/G^∗^-luciferase were collected from the supernatants 24 h post-infection. Infecting the cells with rVSV-ΔG/G^∗^-luciferase pseudovirus caused cell rounding, which was observed in all transfected BHK-21/WI-2 cells after 24 h (Figure 5).

**FIGURE 6:**
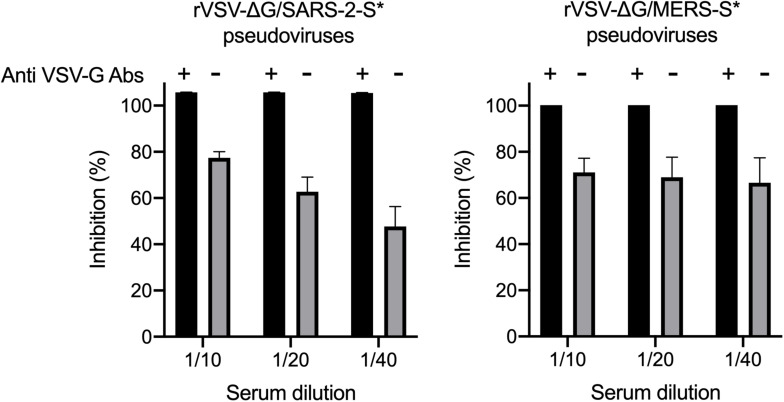
Neutralization of rVSV pseudovirus generated in the absence or presence of anti-VSV-G polyclonal antibodies. Both rVSV-ΔG/MERS-S*-luciferase and rVSV-ΔG/SARS-2-S*-luciferase were generated in the absence or presence of anti-VSV-G polyclonal antibodies and used in neutralization assay using seropositive serum samples in Vero E6 cells. Using anti-VSV-G polyclonal antibodies always resulted in pseudoviruses without residual rVSV-ΔG/G*-luciferase as shown by the complete inhibition of the luciferase activity compared with the partial inhibition when pseudoviruses were generated in absence of anti-VSV-G polyclonal antibodies, which indicated activities from residual rVSV-ΔG/G*-luciferase. Samples were run in duplicates, and data are shown as mean ± SD from one representative experiment out of three independent experiments. Inhibition (%) was calculated as 100 - [(mean RLU from each sample (virus + diluted serum) - mean RLU from CC)/(mean RLU from VC - mean RLU from CC) × 100]. rVSV, recombinant vesicular stomatitis virus; RLU, relative luminescence unit; CC, cell-only control.

One of the most common problems that could occur during the generation of pseudoviruses is having residual or background rVSV-ΔG/G^∗^-luciferase pseudovirus in the collected supernatants (Nie et al., 2020). Complete removal of excess rVSV-ΔG/G^∗^-luciferase that do not infect the cells was done by washing the cell monolayer twice with 1 × PBS as well as using DMEM-5 supplemented with anti-VSV-G antibodies. As shown in Figure 6, complete inhibition of the luciferase activity by seropositive serum samples was observed in all pseudoviruses (rVSV-ΔG/MERS-S^∗^-luciferase and rVSV-ΔG/SARS-2-S^∗^-luciferase) generated in presence of anti-VSV-G polyclonal antibodies as compared with the partial inhibition of pseudoviruses that were generated in the absence anti-VSV-G polyclonal antibodies. While we used 1:1,000 dilution of anti-VSV-G polyclonal antibodies and found it effective in inhibiting residual rVSV-ΔG/G^∗^-luciferase viruses, dilution of in-house produced anti-VSV-G polyclonal antibodies is dependent on the antibody titer and should be optimized.

### Titration of the Generated rVSV-ΔG/MERS-S^∗^-Luciferase and rVSV-ΔG/SARS-2-S^∗^-Luciferase Pseudoviruses

**FIGURE 7:**
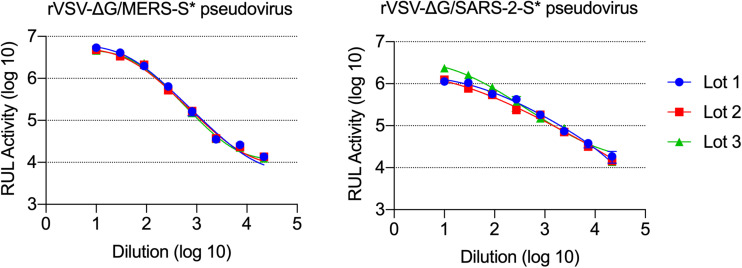
Titration of rVSV-ΔG/MERS-S*-luciferase and rVSV-ΔG/SARS-2-S*-luciferase pseudoviruses. Generated viruses were serially diluted in a 0.5 log dilution and used to titrate luciferase activity in Vero E6 cells. Luciferase activity was plotted against each dilution using 4PL logistic curve. Samples were run in duplicates, and data are shown as mean ± SD. Data are shown from three different production lots of rVSV-ΔG/MERS-S*-luciferase and rVSV-ΔG/SARS-2-S*-luciferase pseudoviruses. rVSV, recombinant vesicular stomatitis virus.

Measuring titers of the produced pseudoviruses was based on luminescence signal reads obtained from the activity of the expressed luciferase. Vero E6 cells were selected for pseudovirus titration as they are permissive for MERS-CoV, SARS-CoV-2, and VSV. After 24 h of infecting the seeded Vero E6 cells in 96-well white or black plate with clear bottom with serial dilution of generated pseudoviruses, the cells were lysed and the luciferase activities were measured. The measured luciferase activity is defined as RLU. Based on the RLU values observed at each serial dilution, virus dilution vs. RLU readout was plotted to select the needed amount of virus for further experiments (Figure 7). The right titer of the generated pseudoviruses was selected as the dilution that results in an RLU above cell-only control and in the linear part of the curve (1 × 10^4^–5 × 10^5^). In our case, we used ∼5 × 10^4^ RLU/well to maximize the use of each lot of the produced pseudoviruses, and it worked very well in neutralization assays. Figure 7 also shows the activity from different lots of each produced pseudovirus (rVSV-ΔG/MERS-S^∗^-luciferase and rVSV-ΔG/SARS-2-S^∗^-luciferase pseudoviruses), which shows minimum variation between the different lots.

### Neutralization Assay With rVSV-ΔG/MERS-S^∗^-Luciferase and rVSV-ΔG/SARS-2-S^∗^-Luciferase Pseudoviruses

**FIGURE 8:**
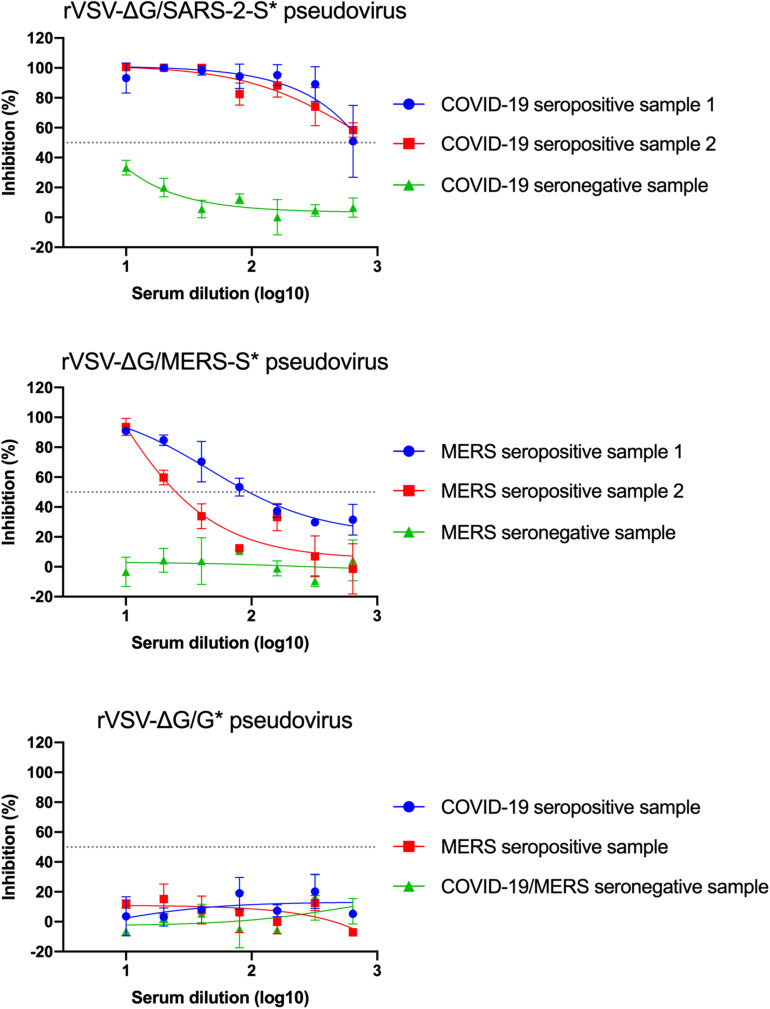
Example of neutralization activity using the generated pseudoviruses. Inhibition of rVSV-ΔG/MERS-S*-luciferase, rVSV-ΔG/SARS-2-S*-luciferase, or rVSV-ΔG/G*-luciferase pseudoviruses using seropositive and seronegative human serum samples in Vero E6 cells. Data were plotted using 4PL logistic curves as indicated in section “Materials and Methods.” Serum samples were diluted starting from 1:10 to 1:640. Samples were run in duplicates, and data are shown as mean ± SD from one representative experiment out of three independent experiments. rVSV, recombinant vesicular stomatitis virus.

Determination of nAb titers in serum samples depends on measuring the inhibition level of luciferase activity from pseudoviruses in cells, which is correlated with the inhibition of virus entry into Vero E6 cells. Twofold serial dilutions of seropositive serum samples from confirmed MERS-CoV and SARS-CoV-2 infected patients were prepared and pre-incubated with 5 × 10^4^ RLU of rVSV-ΔG/MERS-S^∗^-luciferase and rVSV-ΔG/SARS-2-S^∗^-luciferase pseudoviruses, respectively. Sera from healthy donor were included as a negative control. As shown in Figure 8, dose-dependent inhibition in luciferase activity was observed with tested seropositive serum samples, in contrast to the seronegative control sera, which did not show any significant changes in the luciferase activity at any dilution. These results demonstrated the both generated pseudoviruses specially neutralized by corresponding seropositive serum samples. As expected, testing these serum samples against rVSV-ΔG/G^∗^-luciferase pseudovirus showed no inhibition (Figure 8). Neutralizing Ab titer could be computed as IC_50_ using 4PL logistic curve in graph prism or similar software. Otherwise, relative 50% nAb titers (NT_50_) in seropositive serum samples could be determined as the reciprocal of the highest serum dilution that reduces luciferase activity by 50% relative to VC only.

## Discussion

We have used the rVSV-ΔG/G^∗^-luciferase system to measure nAbs against MERS-CoV and SARS-CoV-2 in serum samples. The detailed protocols described here include all the steps are needed to generate rVSV pseudoviruses to implement neutralization assay for highly pathogenic CoVs in BSL-2 laboratories. The availability of such protocol would help different laboratories and researchers to study the seroprevalence of CoVs in the population, to study immune response in infected individuals, and to evaluate vaccine immunogenicity and viral entry inhibitors (Fukushi et al., 2005; Lester et al., 2019; Almasaud et al., 2020; Nie et al., 2020). By following our protocol, generation and titration of desired pseudoviruses can be performed easily and quickly using basic techniques and equipment in BSL-2 environment. Pseudovirus neutralization assay takes 3 days, and up to six serum samples could be tested per plate. In our work, we succeed to generate pseudovirus stocks without having them contaminated with residual rVSV-ΔG/G^∗^-luciferase viruses that did not infect cells. We found that adding anti-VSV-G antibodies has improved the neutralization assay by eliminating high background readings of luciferase activity that could lead to false-negative results. While we used full-length S gene from SARS-CoV-2 to generate good titers of rVSV-ΔG/SARS-2-S^∗^-luciferase to perform the neuralization assays, it has recently been reported that deletion of 21 amino acid at the cytoplasmic tail of the S protein from SARS-CoV-2 could enhance the titer significantly (Case et al., 2020). The work presented here is reproducible and allows testing human or animal sera without using live viruses in BSL-3 bio-containment laboratories.

## Data Availability Statement

All datasets generated for this study are included in the article/supplementary material.

## Author Contributions

SA, AA, M-ZE, and AH performed the methodology and testing. SA, AA, MA, and AH wrote the original draft. SA, AA, MA, M-ZE, and AH wrote, reviewed, and edited the manuscript. All authors contributed to the article and approved the submitted version.

## Conflict of Interest

The authors declare that the research was conducted in the absence of any commercial or financial relationships that could be construed as a potential conflict of interest.
